# Losing the rose tinted glasses: neural substrates of unbiased belief updating in depression

**DOI:** 10.3389/fnhum.2014.00639

**Published:** 2014-08-28

**Authors:** Neil Garrett, Tali Sharot, Paul Faulkner, Christoph W. Korn, Jonathan P. Roiser, Raymond J. Dolan

**Affiliations:** ^1^Affective Brain Lab, Experimental Psychology, University College LondonLondon, UK; ^2^Institute of Cognitive Neuroscience, University College LondonLondon, UK; ^3^Department of Psychiatry, Psychotherapy, and Psychosomatics, Comparative Emotion Group, University of ZurichZurich, Switzerland; ^4^Wellcome Trust Center for Neuroimaging, Institute of Neurology, University College LondonLondon, UK

**Keywords:** depression, decision making, belief updating, optimism, biased processing

## Abstract

Recent evidence suggests that a state of good mental health is associated with biased processing of information that supports a positively skewed view of the future. Depression, on the other hand, is associated with unbiased processing of such information. Here, we use brain imaging in conjunction with a belief update task administered to clinically depressed patients and healthy controls to characterize brain activity that supports unbiased belief updating in clinically depressed individuals. Our results reveal that unbiased belief updating in depression is mediated by strong neural coding of estimation errors in response to both good news (in left inferior frontal gyrus and bilateral superior frontal gyrus) and bad news (in right inferior parietal lobule and right inferior frontal gyrus) regarding the future. In contrast, intact mental health was linked to a relatively attenuated neural coding of bad news about the future. These findings identify a neural substrate mediating the breakdown of biased updating in major depression disorder, which may be essential for mental health.

## INTRODUCTION

Contrary to traditional psychological theories that maintain that good mental health is sub-served by accurate beliefs in relation to reality (Maslow, 1950; Jahoda, 1958; Erikson, 1963), a large body of literature (Allport, 1955; Taylor and Brown, 1988) suggest biases may promote adaptive functioning. In particular, “positive illusions” (including overly positive evaluation of the self, unrealistic optimism and an exaggerated sense of control) are argued to enhance mental health by encouraging productivity, social interaction, subjective happiness and physical health (Taylor et al., 2003; McKay and Dennett, 2010); but see Compton (1992), Colvin and Block (1994). In healthy individuals, positive illusions are especially apparent under circumstances of adversity (Taylor and Armor, 1996) which may enhance resiliency to stressful life events. By contrast, moderately depressed individuals have been reported to display a less positive, but relatively unbiased, view of the self (Coyne and Gotlib, 1983), the future (Strunk and Adler, 2009), and sense of control (Alloy and Abramson, 1979) – dubbed “depressive realism” [but see Allan et al. (2007) and Moore and Fresco (2007)]. Importantly, however, severely depressed individuals often show negative biases in these domains (Roiser et al., 2012) which can predict fatal outcomes (Oquendo et al., 2004).

Positive biases are generated in healthy individuals because while people incorporate desirable information into existing beliefs according to Bayes’ Rule (Eil and Rao, 2011), for undesirable information they show an aversion to incorporate new information (Köszegi, 2006) and discount its impact (Eil and Rao, 2011). We recently found that depressed patients lack the positive skewed belief updating bias seen in healthy controls when they update their expectations about the future (Korn et al., 2013). These findings raise an intriguing question as to what differs in depression in terms of underlying neural substrates that support an unbiased belief formation about the future.

We have previously demonstrated that, in healthy participants, biased updating in response to positive and negative news is mediated by a relatively weak correlation between brain activity and negative estimation errors, but intact coding of positive estimation errors (Sharot et al., 2011). Here, utilizing the belief updating task in combination with functional brain imaging we ask whether depression is associated with neural responses that are likely to support a more unbiased integration of information about the future

## MATERIALS AND METHODS

### PARTICIPANTS

Thirty individuals aged 18–65 participated in the study (half unmedicated depressed patients and half healthy controls). Depressed participants were identified through Camden and Islington Foundation Trust Psychological Treatment Services or recruited by advertisement. Healthy controls were recruited from UCL psychology subject pool and matched to depressed participants for age, gender, and level of education. One control participant was subsequently excluded from the analysis due to a high score on the Beck Depression Inventory (>10). None of the participants had taken antidepressant medication for at least 6 weeks prior to undertaking the study due to a variety of personal choices unrelated to the study itself. No participants had a period of substance or alcohol abuse in the 6 months prior to undertaking the study. See **Table 1** for demographic and clinical information.

**Table 1:** Demographic and clinical information of participants.

	Controls	MDD patients
*N* (Male)	14 (9)	15 (9)
Age^1^	30.36 (8.35)	31.47 (9.16)
Level of education*^2^	2.64 (0.81)	2.60 (0.88)
BDI^3^	2.21 (2.75)	25.80 (9.97)
*N* with history of alcohol/substance abuse	0 (0%)	4 (26%)
*N* receiving psychotherapy	0 (0%)	2 (13%)
Age of onset of first depressive episode	0 (0%)	18.64 (7.01)
*N* with at least two depressive episodes	0 (0%)	14 (93%)
*N* previously attempting suicide	0 (0%)	2 (13%)

Figures represent mean (SD) unless stated otherwise.

*Coded as follows: (1) High School; (2) Bachelors Degree; (3) Masters Degree; (4) Ph.D.

*^1^Independent sample *t-*test: *t*(27) = 0.34 ( >0.73).*

*^2^Independent sample *t-*test: *t*(27) = -0.13 ( >0.89).*

*^3^Independent sample *t-*test: *t*(27) = 8.71 ( <0.05).*

Before the study, all participants were assessed for psychiatric disorders by a trained researcher using the Mini International Neuropsychiatric Inventory (MINI; Sheehan et al., 1998). Training involved the researcher observing six separate MINI interviews and then being observed carrying out four MINI interviews with detailed feedback provided after each interview. The MINI confirmed that depressed participants had experienced depressive episodes in the past and met criteria for a major depressive episode at the time of undertaking the study. For controls, the MINI confirmed that participants had not experienced any depressive episodes during their lifetime. The MINI was also used to verify that participants in both groups had no other past or present psychiatric conditions, other than anxiety disorders in the depressed participants. Participants that did not conform to any of the above were not invited to participate in the study further. All participants completed the Beck Depression Inventory (Beck et al., 1961) controls mean = 2.21 (range: 0–9), MDD mean = 25.80 (range 13–44). The majority of depressed participants were mildly (*n* = 5) or moderately (*n* = 7) depressed, with a minority (*n* = 3) severely depressed.

All participants gave informed consent and were paid for their participation. The study was approved by the London Queen Square Research Ethics Committee.

### LIFE EVENTS

Eighty short descriptions of negative life events (e.g., passenger in a car accident, home burglary – see Sharot et al., 2011) were presented in random order. For each adverse event the average probability of that event occurring at least once to a person living in the same socio-cultural environment as the participants was determined from online resources (Office for National Statistics, Eurostat, PubMed). Very rare, or very common, events were not included; all events probabilities lay between 10 and 70%. To ensure that the range of possible overestimation was equal to the range of possible underestimation, participants were told that the range of probabilities lay between 3 and 77%.

### PROCEDURE

The procedure was identical to our previous study (Sharot et al., 2011), and we summarize it below. Participants went through three practice trials. The session began with a short structural scan, followed by four functional runs consisting of 40 trials each (all 80 events were presented twice). Finally, an additional longer structural scan was performed.

### BEHAVIORAL TASK

**FIGURE 1:**
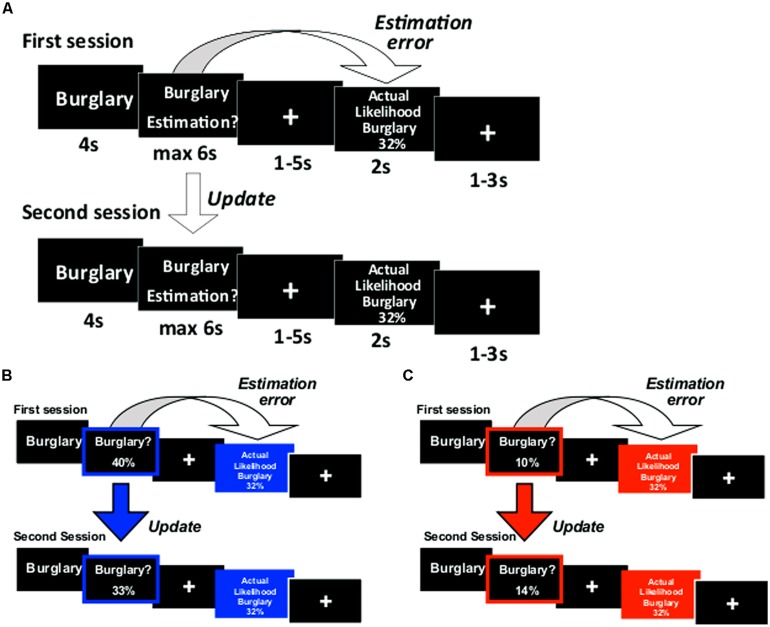
**Paradigm. (A)** On each trial participants were presented with a short description of one of 80 adverse events and asked to estimate how likely this event was to occur to them. They were then presented with the average probability of that event occurring to a person living in the same sociocultural environment. The second session was the same as the first. For each event an update term was calculated as the difference between the participant’s first and second estimations. Examples of trials for which the participant’s estimate was **(B)** higher or **(C)** lower than the average probability. Here, for illustration purposes only, the blue and red frames denote the participant’s response (either an overestimation or underestimation, respectively) and the blue and red filled boxes denote information that calls for an adjustment in a **(B)** desirable (good news) or **(C)** undesirable (bad news) direction.

The paradigm was adapted from our previous studies (Sharot et al., 2011, 2012a,b; Chowdhury et al., 2013; Korn et al., 2013; Moutsiana et al., 2013) and depicted in **Figure 1**. On each trial a life event was presented on screen for 4 s. Participants were instructed to think of that event happening to them in the future. After 4 s, participants were to respond in the following manner: in half of the runs (either runs 1 and 2 or runs 3 and 4, counterbalanced across participants), the words “Estimation of happening?” appeared on screen and participants entered their estimated likelihood of the event happening to them in the future. In the other two runs, the words “Estimation of NOT happening?” appeared on screen and participants entered their estimated likelihood of the event not happening to them in the future. We framed estimations in these two ways so that (1) differential processing of good news and bad news (that is, overestimation and underestimation of the likelihood of an event) could not be attributed to differential processing of high and low numbers (2) half the life events would be framed as negative (getting robbed) and half positive (never getting robbed), yet the information received in both cases could be good (less likely to get robbed, more likely never to get robbed) or bad (more likely to get robbed, less likely never to get robbed). If participants had already experienced an event in their lifetime they were instructed to estimate the likelihood of that event happening (or not happening) to them again in the future.

Participants had up to 6 s to respond using a button box with four buttons in each hand. Each button corresponded to one digit. The digits 0 through 7 could be used to enter the estimated likelihoods in the “happen” estimation and digits 2 through 9 in the “not happen” estimation. If the participant failed to respond, then that trial was excluded from all subsequent analyses (mean trials with no response = 1.38, SD = 2.83). A fixation cross then appeared for 1–5 s (jittered). Next, the event description appeared again for 2 s, together with the average probability of that event to occur (or not occur, depending on “happen” or “not happen” sessions). Finally, a fixation cross appeared for 1–3 s (jittered).

We were interested in how participants altered their beliefs in response to the information given. Thus, participants estimated each event twice in two consecutive sessions (before and after they received information). One list of 40 life events (counterbalanced) was presented during scan 1 and then again during scan 2. The other list was presented during scan 3 and then again during scan 4.

To test participants’ memory for the information presented we asked participants, after the scanning sessions, to provide the actual probability previously presented of each event. Participants then rated all life events on: vividness (“How vividly could you imagine this event?” From 1 = not vivid to 6 = very vivid); familiarity (“Regardless if this event has happened to you before, how familiar do you feel it is to you from TV, friends, movies and so on?” From 1 = not at all familiar to 6 very familiar); prior experience (“Has this event happened to you before?” From 1 = never to 6 = very often); arousal (“When you imagine this event happening to you how emotionally arousing is the image in your mind?” From 1 = not arousing at all to 6 = very arousing) and negativity (“How negative would this event be for you?” From 1 = not negative at all to 6 = very negative).

### BEHAVIORAL ANALYSIS

Behavioral analysis was conducted as described previously (Sharot et al., 2011, 2012a,b; Chowdhury et al., 2013; Korn et al., 2013; Moutsiana et al., 2013) using IBM SPSS statistics (version 19). All actual (statistical) and estimated percentages in the “not happen” sessions were transformed into the corresponding numbers of the “happen” sessions by subtracting the respective number from 100. For each participant, trials were classified according to whether the participant initially overestimated or underestimated the probability of the life event relative to the average probability presented. Specifically, if their initial estimate was lower than the average presented, this information would be categorized as “bad news.” If their initial estimate was higher than the average presented, this information would be categorized as “good news.” Trials in which the initial estimate was equal to the average presented were excluded from subsequent analyses (mean = 1.83 trials, SD = 1.65) as these could not be categorized into either condition.

For each event in each session, an estimation error term was calculated as the absolute difference between the participant’s estimate and the corresponding statistical probability presented:

Estimation Error = | First Estimate – Probability Presented |

Update was calculated as follows:

Update (Good News) = First Estimate – Second Estimate

Update (Bad News) = Second Estimate – First Estimate

Thus, positive updates indicate a change toward the probability presented and negative updates a change away from the probability presented. Average update scores were then entered into a two (valence: good/bad) by two (group: MDD/Control) repeated-measures ANOVA.

To explore the relationship between estimation errors and update, for each participant, two linear regressions were conducted entering estimation errors as independent measures and updates as dependent measures – one for trials in which participants received good news and one for trials in which participants received bad news. Thus, we defined two learning scores for each participant (one for good and one for bad news) as the regression coefficients corresponding to the slope in each regression.

Memory errors were calculated as the absolute difference between the probability previously presented and the participants’ recollection of that statistic:

Memory Error = | Actual Probability Presented – Recollection of Probability Presented |

Average memory error scores for good news and bad news were calculated for each participant and entered into a two (valence: good/bad) by two (group: MDD/Control) repeated-measures ANOVA. ANOVAs were also performed on scores of all other scales (negativity, emotional arousal, vividness, familiarity, past experience) as well as on other task measures (initial estimates, reaction times, estimation errors, number of trials).

### MRI SCANNING

Scanning was performed at the Wellcome Trust Center for Neuroimaging at UCL using a 3T Siemens Allegra scanner with a Siemens head coil. Functional images were acquired as echo-planar (EPI) T2^∗^-weighted images. Time of repetition (TR) = 2.73 s, time of echo (TE) = 30 ms, flip angle (FA) = 90, matrix = 64 × 64, field of view (FOV) = 192 mm, slice thickness = 2 mm. A total of 42 axial slices (-30°tilt) were sampled for whole brain coverage, in-plane resolution = 3 × 3 mm.

### fMRI DATA ANALYSIS

Statistical Parametric Mapping (SPM5, Wellcome Trust Center for Neuroimaging^1^) was used for fMRI data analysis. After discarding the first six dummy volumes, images were realigned to the seventh volume, unwarped, normalized to a standard EPI template based on the Montreal Neurological Institute (MNI) reference brain, resampled to 2 mm × 2 mm × 2 mm voxels and spatially smoothed with an isotropic 8 mm full-width at half-maximum Gaussian kernel. Low frequency artifacts were removed using a 1/128 Hz high-pass filter and temporal autocorrelation intrinsic to the fMRI time series was corrected using an AR(1) process.

For each participant, we created a design-matrix with event onsets time-locked to the temporal positions of: event presentation; presentation of cue prompting response; motor response; and presentation of information. These were modeled as durations of 4, 0, and 2 s, respectively. For all task components (except for motor responses), regressors were subdivided into two conditions: trials of events for which participants received good news and trials of events for which they received bad news, resulting in seven regressors for each session. These events were convolved with a canonical hemodynamic response function (HRF) to create regressors of interest. Motion correction regressors estimated from the realignment procedure were entered as covariates of no interest.

To identify regions tracking estimation errors, we entered absolute estimation errors as parametric regressors modulating the events in which information was presented. For each condition (that is, for trials in which information was better than expected and trials in which information was worse) we identified regions showing significant effects across *both* healthy and depressed participants (*p* < 0.05, cluster level corrected across the whole brain; images first thresholded at *p* < 0.001, uncorrected). Owing to the fact that past research (Sharot et al., 2011) showed that individual differences in our task are best predicted by region(s) inversely tracking bad news estimation errors, betas from the peak voxel in the region(s) tracking bad news estimation errors were extracted and compared between depressed participants and controls using an independent sample *t-*test (*p* < 0.05) in SPSS. Previous research suggests that peak voxel activity can be a better predictor of electrophysiological measures of activation than average cluster (Arthurs and Boniface, 2003).

In addition, the right inferior frontal gyrus (rIFG) was an a-priori ROI because our previous findings (Sharot et al., 2011) showed that the degree to which BOLD signals in this region track bad news estimation errors differentiated between participants with high and low trait optimism. Thus we examined whether a similar difference existed between depressed and controls in the anatomically defined rIFG using small volume correction.

All activations are displayed on sections of the standard MNI reference brain. Anatomical labels were assigned using the Talairach Daemon database (University of Texas Health Science Center San Antonio^2^) according to peak voxels in Talairach and Tournoux coordinate space. rIFG was anatomically defined by creating an ROI mask using WFU Pickatlas^3^.

## RESULTS

### UNBIASED UPDATING IN MDD, BUT BIASED UPDATING IN CONTROLS

**FIGURE 2:**
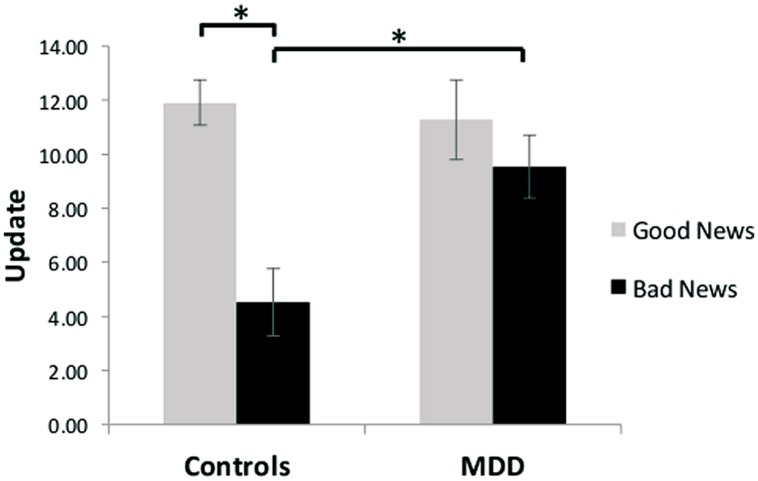
**Unbiased updating in MDD, but biased updating in controls.** After receiving good news that presented an opportunity to adjust beliefs in a positive direction, healthy participants updated their estimations to a greater extent than after receiving bad news that called for adjustments in a negative direction. In contrast, depressed participants updated their beliefs to a similar extent after receiving good and bad news, and updated their beliefs more than healthy individuals when receiving bad news. Error bars represent SEM *p < 0.05, two-tailed independent/paired samples *t*-test.

Our results revealed unbiased updating in depressed individuals, but a valance-dependent updating bias in healthy individuals. Specifically, a group (MDD/healthy) by valence (good news/bad news) ANOVA revealed a significant interaction [*F*(1,27) = 9.16, *p* < 0.01, **Figure 2**]. Replicating previous findings (Sharot et al., 2011, 2012a,b; Chowdhury et al., 2013; Korn et al., 2013; Moutsiana et al., 2013), we show that healthy participants updated their beliefs to a greater extent in response to good news relative to bad news [*t*(13) = 5.00, *p* < 0.001; 93% of healthy participants showed greater updating in response to good news]. No such difference was observed in the MDD group [*t*(14) = 1.49, *p* > 0.15; 60% of depressed participants showed greater updating in response to good news, see also Korn et al. (2013) for similar findings in hospitalized, medicated, depressed patients]. The interaction was further characterized by greater updating in response to bad news [*t*(27) = 2.96, *p* < 0.01] in the MDD group compared to the healthy controls, with no significant difference in updating between groups in response to good news [*t*(27) = -0.37, *p* > 0.70]. Our results suggest that depression, in contrast to good mental health, is related to a lack of discounting of bad news, resulting in unbiased updating of beliefs in response to good and bad news.

### DIFFERENTIAL UPDATING IN HEALTH AND DISEASE CANNOT BE EXPLAINED BY MEMORY, FAMILIARITY, PAST EXPERIENCE WITH THE AVERSIVE LIFE EVENTS, VIVIDNESS, REPORTED AROUSAL, REPORTED NEGATIVITY, REACTION TIMES, PRIORS, NUMBER OF TRIALS

To examine whether the relationship between depression and updating could be explained by any other factor, we tested for a relationship between depression and all other variables recorded. Note, that we do not perform corrections for multiple comparisons because the aim of these analyses was to identify potential confounding factors; thus, by not using Bonferroni corrections, our analyses are more stringent.

#### Memory

After the scanning session, participants were asked to indicate the actual probability (as previously presented) of each event occurring on average. Memory errors were calculated as the absolute difference between the actual probability previously presented and the participants’ recollection of that statistic. Memory errors did not differ between groups (see **Table 2**) and there was no interaction with valence [*F*(1,27) = 1.07, *p* = 0.31].

**Table 2:** Participants’ ratings of familiarity with stimuli, prior experience, vividness, arousal, negativity, memory, initial estimates, reaction times and number of trials.

	MDD mean (SD)		CONTROLS mean (SD)	
Questionnaire and variables	Good news	Bad news	Good news	Bad news
***Subjective Scales Questionnaire: all scales 1 = low to 6 = high***				
Familiarity^v^	3.88 (0.82)	3.62 (0.84)	4.06 (0.99)	3.67 (0.92)
Prior experience^v^	1.65 (0.31)	1.46 (0.24)	1.50 (0.68)	1.39 (0.64)
Vividness^v^	3.87 (0.84)	3.52 (0.75)	3.68 (0.89)	3.50 (0.81)
Emotional arousal	3.24 (0.79)	2.95 (0.70)	3.29 (0.85)	3.34 (0.95)
Negativity ^v∗g^	3.97 (0.71)	3.76 (0.61)	3.98 (0.68)	4.09 (0.67)
***Task-related variables***				
Memory errors	11.26 (4.37)	10.39 (3.38)	11.23 (3.29)	11.45 (1.86)
Initial estimates^v^	43.25 (7.35)	21.01 (3.2)	44.07 (5.22)	19.84 (4.47)
Reaction time first estimate (ms)	2183.01 (663.41)	2153.83 (578.97)	2298.73 (790.90)	2303.33 (716.04)
Reaction time second estimate (ms)^v∗g^	1762.00 (533.63)	1819.19 (566.46)	1997.20 (648.74)	1891.72 (618.93)
Number of trials	36.53(12.44)	34.67(8.55)	34.64(12.30)	36.71(7.04)

*^v^Main effect valence, *p* < 0.05.*

*^g^Main effect group, *p* < 0.05 (NB: there was no group difference for any of the variables).*

*^v∗g^Interaction effect (group × valence), *p* < 0.05.*

#### Life event ratings

Participants rated life events on five scales (past life experience with the events, familiarity with the events, ability to imagine them vividly, emotional arousal and negativity). The scores revealed that past experience and familiarity with the adverse life events, as well as the ability to vividly imagine the events and the subjective sense of emotional arousal in response to the events, *did not differ* between MDD and healthy controls and did not interact with valence (**Table 2**). However, how negative the participants rated the events did reveal a group by valence interaction [*F*(1,27) = 6.76, *p* = 0.02]. MDD patients rated life events that they received bad news for during the experiment as less aversive than events they received good news for. This was the opposite in the case of healthy controls who rated life events for which they received bad news as more aversive than life events for which they received good news. Thus we repeated the main analysis of update scores while controlling for differential scores of negativity (i.e., scores on good news trials minus scores on bad news trials). After entering these scores as covariates, the group by valance interaction on update scores remained significant [*F*(1,26) = 5.10, *p* < 0.05]. Thus, differential update *could not* be explained by differences in the degree of the perceived negativity of the events, by familiarity or by past experience with the events, by whether the events were imagined vividly or experienced as more or less emotionally arousing.

#### Task factors (number of trials, priors, estimation errors, RTs)

There were no differences across groups in the number of missed responses, nor the number of good news and bad news trials. These factors did not differ across valence nor did valence interact with group (see **Table 2** for statistics). Thus, MDD participants did not miss more responses than controls and were not more likely to encounter good news trials than bad news trials. The magnitude of the estimation errors did not differ between groups [bad news: *t*(27) = -0.02, *p*> 0.99, good news *t*(27) = 0.64, *p*> 0.53]. Participants’ priors – their initial estimates of the probability of the events – did not differ across groups, however, they did correlate with BDI scores (*r* = 0.38; *p* < 0.05). In other words, the more depressed the individual, the more likely they were to estimate their chances of encountering aversive events as greater (also see Strunk et al., 2006). Reaction time for first estimates did not differ between groups. However, reaction time for second estimates did reveal a valence by group interaction [*F*(1,27) = 6.24, *p* < 0.05] with MDD participants slightly faster than controls to re-estimate their likelihood of encountering an event they previously received good news for. After entering the difference in second estimate reaction time as a covariate along with initial estimates and differential scores of negativity (see above), the group by valance interaction on update scores remained significant [*F*(1,24) = 5.39, *p* < 0.05].

#### Framing

Whether participants were asked to estimate the likelihood of the events happening in the future, or never happening, did not alter our results. A group (MDD, healthy) by valence (good news/bad news) by frame (happen/not happen) ANOVA revealed the expected two-way interaction of group by valence [*F*(1,27) = 4.92, *p* < 0.04], which is driven by controls updating more on good news trials than bad and MDD showing unbiased updating. However, there were no other significant interactions with group. Note, that reaction times did not differ between frames [*t*(28) = 0.96, *p* > 0.34] nor groups [*t*(28) = 1.09, *p* > 0.28] nor was there an interaction between group and frame [*F*(1,27) = 0.131, *p* > 0.7].

### UNBIASED UPDATING IN MDD EXPLAINED BY ADEQUATE USE AND NEURAL TRACKING OF NEGATIVE ESTIMATION ERRORS

#### Learning scores

**FIGURE 3:**
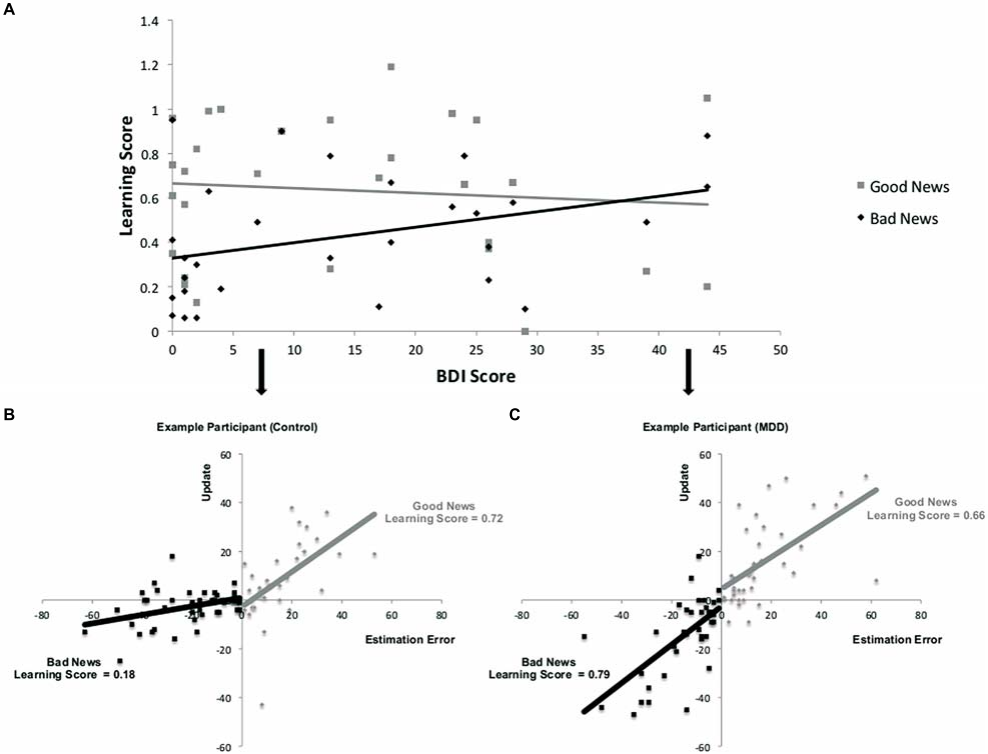
**Relationship between depression and learning from good and bad news. (A)** Correlation across participants between BDI and learning scores from good and bad news. **(A,B)** Learning is defined here as the relationship between estimation errors and update across trials for each subject. Data from two participants demonstrate this association for trials in which the participant received good news and trials in which the participant received bad news. The slope of each line is the learning score of that participant. In this example, learning from bad news is worse than learning from good news in the healthy participant **(B)** but does not differ as much for the depressed participant **(C)**. Note that for display purposes we have reversed the sign of update and estimation errors for bad news trials so that the plots do not sit on top of each other.

As depicted in **Figures 3B,C**, learning scores are calculated by quantifying the relationship, on a trial-by-trial basis for each participant between an estimation error and subsequent update. The resulting regression coefficient indexes the learning score (note that this is different from the update score discussed in the previous section). While participants learned from the information presented to them [mean regression coefficients relating an individual’s estimation errors to update was significantly different from zero; *t*(28) = 10.79, *p* < 0.001], their ability to do so was differentially related to depression symptoms as a function of valence. Specifically, the more depressed the participant, as indicated by BDI score, the greater the learning score was for bad news, but learning scores for good news were not reliably associated with depression (**Figure 3A**). This was evident in a positive correlation between BDI and learning scores in trials when participants received bad news (*r* = 0.36, *p* = 0.05) and no correlation between BDI and learning scores when receiving good news (*r* = -0.09, *p* > 0.62). The difference between these two correlations was statistically significant (*Z* = 2.17, *p* < 0.05, Steiger’s *Z* test).

Our findings suggest a likely computational principle that mediates the observed unbiased belief formation in depression. Specifically, they point to estimation errors as providing a learning signal whose impact on update depends on an interaction between depressed mental state and whether this new information calls for an update in a positive or negative direction.

#### fMRI data

Given the above results we examined our fMRI data to identify how BOLD signals track estimation errors in response to information that entails a belief adjustment in either a positive or a negative direction in depressed and healthy individuals. Absolute estimation errors on each trial were entered as a parametric regressor modulating the time point at which participants were presented with information regarding the average probability of events. From this analysis, we first identified regions where BOLD signal correlated with estimation errors for either good or bad news on a trial by trial basis across *all* participants

**FIGURE 4:**
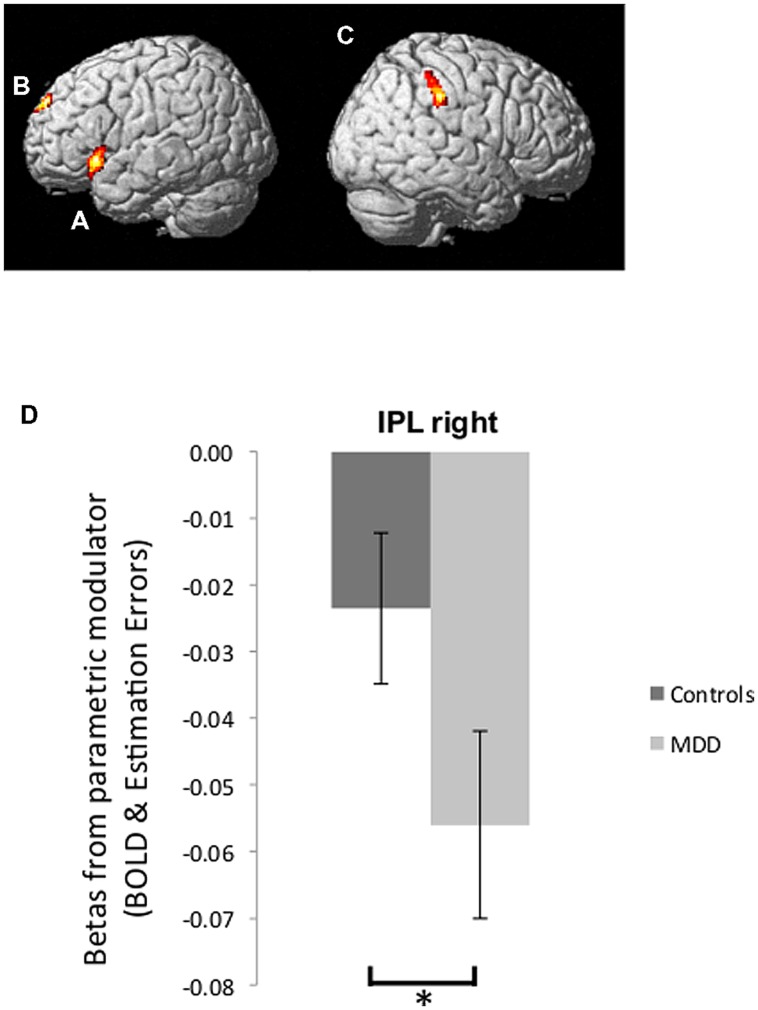
**Brain activity tracking estimation errors. (A,B)** Regions in which BOLD signal tracked participants’ estimation errors on a trial-by-trial basis across both groups in response to good news regarding future likelihoods included the left IFG **(A)** and bilateral SFG **(B)** (*p* < 0.05, FWE cluster level corrected). **(C)** BOLD signal tracking participants’ estimation errors in response to bad news was found in the right IPL (*p* < 0.05, FWE cluster level corrected). **(D)** Parameter estimates of the parametric regressors from peak voxels in the right IPL showed a stronger inverse correlation between BOLD activity and bad news errors in depressed individuals relative to healthy individuals. Error bars represent SEM *p < 0.05, two-tailed independent samples *t*-test.

BOLD signal correlated positively with good news estimation errors in the left inferior frontal gyrus (left IFG: peak voxel in Talairach coordinates: –50, 17, –4; *k* = 293; *z* = 4.31, **Figure 4A**) and bilateral superior frontal gyrus (bilateral SFG: -6, 60, 26; *k* = 174; *z* = 4.01, **Figure 4B**). In addition BOLD signal correlated negatively with bad news estimation errors in the right inferior parietal lobule (right IPL: 65, -27, 36; *k* = 185; *z* = 4.30, **Figure 4C**) and positively with bad news errors in Superior Temporal Gyrus (-44, -50, 14; *k* = 1174; *z* = 4.92) and Superior Frontal Gyrus (-2, 50, 31; *k* = 203; *z* = 3.80). There were no voxels in which activity correlated negatively with good news estimation errors. These five ROIs comprise the entire set of regions identified in the current dataset at this threshold (FWE cluster level corrected after voxel-wise thresholding at *p* < 0.001)

We then examined whether the extent to which brain activity tracked estimation errors was related to depression. We were specifically interested in the region where BOLD signal was *inversely* tracking bad news estimation errors because our past research (Sharot et al., 2011) showed that this is the pattern of activity that predicts individual differences. We thus compared betas (relating BOLD signal to estimation errors) from the peak voxel in rIPL for participants in the MDD group and control group. Indeed, this revealed that BOLD response in the rIPL of depressed participants tracked bad news errors with greater fidelity than was the case for healthy controls [*t*(27) = -2.27, *p* < 0.05, **Figure 4D**; note that betas are negative, which indicate an inverse correlation – the larger the magnitude of the negative number, the stronger the relationship between BOLD signal and bad news estimation errors].

In addition we tested for differences in the anatomically defined right IFG, as we have previously found that participants with low trait optimism were more likely to have a negative correlation between BOLD signal and bad news estimation errors in this area in the exact same task (Sharot et al., 2011). Indeed, we observed here a stronger negative correlation between BOLD activity in the right Inferior Frontal Gyrus (rIFG) and bad news estimation errors in depressed patients compared to healthy controls [*t*(27) = 4.52, *p* < 0.05 FWE, small volume corrected].

None of the above effects can be explained by the magnitude of the estimation errors – as reported above these did not differ between groups. In other words, rIPL and rIFG representation of errors in response to bad news differentiated depressed individuals from healthy controls. For completeness we tested for differences between groups in the other ROIs identified – none were observed. Together, these results suggest that adequate computational exploitation, and representation of, negative estimation errors in depression underlies a relatively unbiased belief formation.

## DISCUSSION

A substantial body of research now suggests that optimal mental health is associated with unrealistic positive beliefs regarding the self (Taylor and Armor, 1996; Taylor et al., 2003; McKay and Dennett, 2010). According to Bandura (1989), for example, if self-efficacy beliefs were merely to mirror what people could reasonably accomplish, people would seldom fail but neither would they mount the extra effort required to go beyond ordinary performance. If indeed biased beliefs regarding the self are adaptive, there should be a mechanism that promotes formation of such skewed views, one that could be hypothesized to be *unbiased* during maladaptive mental states.

Our results show that clinically depressed participants updated their beliefs in proportion to the error made whether it called for updating in a desirable or undesirable direction, consistent with past results (Korn et al., 2013). In contrast, healthy individuals, were less likely to update beliefs when information called for adjustment in a pessimistic direction. This behavior was mediated by a diminished coding of “bad news” estimation errors in right IPL in healthy individuals, while depression was associated with close coding of negative estimation errors. These results suggest that adequate computational use, and representation of, negative estimation errors in depression underlies a relatively unbiased belief formation. The finding that mild depression may be related in some domains to an absence of a positive bias, rather than a presence of a negative bias also raises an interesting possibility for future research. Namely, testing whether the absence of positively biased belief updating could predict the onset of a depressive episode among individuals at risk for depression.

Interestingly, across groups activity increased for a better than expected outcome in regions tracking positive estimation errors, and dipped for a worse than expected outcome within right IPL. Depressed individuals were also more likely to show a pattern of inverse correlation between BOLD signal and bad news estimation errors in the right IFG than controls. This pattern resembles that of dopaminergic neurons signaling prediction errors (Schultz, 1997). Indeed, we have previously shown that increasing dopamine function (via administration of L-DOPA) enhances an update bias in healthy individuals by impairing updating from negative information even further (Sharot et al., 2012a; see also Frank et al., 2004). Dopamine neurons are known to project to the regions identified here (Fallon and Moore, 1978; Gerfen, 1992; Goldman-Rakic, 2000), and it is of interest that abnormal functioning of the dopaminergic system has been related to depression (Papakostas, 2006) and thus may underlie the *lack* of discounting of negative news observed in the disease.

Our results were *not* explained by how well participants subsequently recalled the information presented to them, as memory for the data provided did not differ across groups. This renders it unlikely that our results are driven by general differences in cognitive or mnemonic abilities. Note also that the participants performed exactly the same task on trials in which they received good news and on trials in which they received bad news, thus valence dependent differences cannot be explained by one group having specific problems with percentages, generally updating less/more or any domain general cognitive function. Neither did our results reflect specific characteristics of events including familiarity with aversive events, how negative the events were perceived to be, how emotionally arousing participants found the events, nor their past life experience with the aversive events. In other words, the depressed participants did not have more experience with these stressful life events and so this cannot explain our results.

Our findings suggest that a positive state of mental health is linked to biased processing and interpretation of information in a manner that supports positively skewed views of the self, while depression is associated with a pattern of activity that supports more unbiased harvesting of information. The data, however, cannot point to causation. Depression may lead to more unbiased updating, or neural systems that supports more unbiased updating of beliefs may generate depression. Furthermore, as depression progresses, and/or in more severe cases, a negatively biased may be observed (the majority of patients in our study were moderately, clinically depressed).

It has been suggested that people create positive life affirming illusions to enable them to cope with uncertainty and anxiety regarding future dire events (Becker, 1997; Varki, 2009). These illusions are particularly apparent under aversive circumstances and promote resilience in such situations (Taylor et al., 1984; Wood et al., 1985). A system that does not allow the creation of such perceptions may promote angst and undermine coping strategies resulting in a downward spiral of the effect of stressful life events on mental health.

## Conflict of Interest Statement

The authors declare that the research was conducted in the absence of any commercial or financial relationships that could be construed as a potential conflict of interest.

## Footnotes

1 http://www.fil.ion.ucl.ac.uk/spm/

2 http://www.talairach.org/

3 http://fmri.wfubmc.edu/software/PickAtlas
